# Supplementary material for: Multi‐dataset identification of innovative feature genes and molecular mechanisms in keratoconus
Source: J Cell Mol Med. 2024 Sep 19;28(18):e70079. doi: 10.1111/jcmm.70079 (PMC11412914; doi:10.1111/jcmm.70079)
Supplement: Supplementary file 2 — Material S2. [file JCMM-28-e70079-s002.zip › 7.GSEA analysis/3.DBNDD1/KEGG_PEROXISOME.html]

Details for gene set KEGG\_PEROXISOME[GSEA]

|  || Dataset | DBNDD1.group.cls#h\_versus\_l |
| Phenotype | group.cls#h\_versus\_l |
| Upregulated in class | h |
| GeneSet | KEGG\_PEROXISOME |
| Enrichment Score (ES) | 0.22545402 |
| Normalized Enrichment Score (NES) | 0.8738053 |
| Nominal p-value | 0.62118125 |
| FDR q-value | 1.0 |
| FWER p-Value | 1.0 |
Table: GSEA Results Summary

  

Fig 1: Enrichment plot: KEGG\_PEROXISOME      
 Profile of the Running ES Score & Positions of GeneSet Members on the Rank Ordered List

  

| SYMBOL | TITLE | RANK IN GENE LIST | RANK METRIC SCORE | RUNNING ES | CORE ENRICHMENT || 1 | FAR2 | na | 36 | 0.707 | 0.1069 | Yes |
| 2 | HAO1 | na | 540 | 0.440 | 0.1596 | Yes |
| 3 | PEX11G | na | 1158 | 0.366 | 0.1977 | Yes |
| 4 | HAO2 | na | 4354 | 0.202 | 0.1363 | Yes |
| 5 | ABCD1 | na | 5834 | 0.155 | 0.1172 | Yes |
| 6 | ABCD4 | na | 7361 | 0.117 | 0.0910 | Yes |
| 7 | HMGCL | na | 7626 | 0.111 | 0.1004 | Yes |
| 8 | CRAT | na | 7691 | 0.110 | 0.1153 | Yes |
| 9 | PEX12 | na | 7838 | 0.107 | 0.1274 | Yes |
| 10 | PEX10 | na | 8283 | 0.099 | 0.1296 | Yes |
| 11 | PEX11A | na | 8287 | 0.099 | 0.1446 | Yes |
| 12 | MVK | na | 8538 | 0.094 | 0.1517 | Yes |
| 13 | PXMP4 | na | 8663 | 0.092 | 0.1621 | Yes |
| 14 | NUDT12 | na | 8679 | 0.091 | 0.1756 | Yes |
| 15 | NUDT19 | na | 8683 | 0.091 | 0.1894 | Yes |
| 16 | ECH1 | na | 9611 | 0.077 | 0.1744 | Yes |
| 17 | ACAA1 | na | 9690 | 0.076 | 0.1837 | Yes |
| 18 | MLYCD | na | 9879 | 0.073 | 0.1894 | Yes |
| 19 | PEX26 | na | 9965 | 0.072 | 0.1979 | Yes |
| 20 | PEX7 | na | 10216 | 0.068 | 0.2011 | Yes |
| 21 | SLC27A2 | na | 10822 | 0.060 | 0.1928 | Yes |
| 22 | ACOX1 | na | 10838 | 0.060 | 0.2016 | Yes |
| 23 | FAR1 | na | 10904 | 0.059 | 0.2087 | Yes |
| 24 | PMVK | na | 10962 | 0.059 | 0.2160 | Yes |
| 25 | PEX5 | na | 11295 | 0.055 | 0.2148 | Yes |
| 26 | PECR | na | 11380 | 0.054 | 0.2206 | Yes |
| 27 | ACOX3 | na | 11488 | 0.052 | 0.2255 | Yes |
| 28 | ACSL1 | na | 12343 | 0.044 | 0.2075 | No |
| 29 | HACL1 | na | 13411 | 0.035 | 0.1820 | No |
| 30 | PEX3 | na | 13491 | 0.034 | 0.1850 | No |
| 31 | ACOT8 | na | 13756 | 0.032 | 0.1823 | No |
| 32 | GNPAT | na | 13784 | 0.032 | 0.1863 | No |
| 33 | PEX16 | na | 14586 | 0.026 | 0.1671 | No |
| 34 | MPV17 | na | 15110 | 0.022 | 0.1554 | No |
| 35 | PEX19 | na | 15493 | 0.019 | 0.1473 | No |
| 36 | PEX11B | na | 16016 | 0.016 | 0.1347 | No |
| 37 | PRDX5 | na | 16066 | 0.015 | 0.1356 | No |
| 38 | EPHX2 | na | 16215 | 0.014 | 0.1335 | No |
| 39 | SLC25A17 | na | 16582 | 0.012 | 0.1248 | No |
| 40 | AGPS | na | 16688 | 0.011 | 0.1235 | No |
| 41 | XDH | na | 16875 | 0.010 | 0.1197 | No |
| 42 | PAOX | na | 16970 | 0.010 | 0.1185 | No |
| 43 | PRDX1 | na | 17213 | 0.008 | 0.1127 | No |
| 44 | SCP2 | na | 17507 | 0.006 | 0.1052 | No |
| 45 | DHRS4 | na | 17878 | 0.004 | 0.0951 | No |
| 46 | BAAT | na | 18325 | 0.001 | 0.0823 | No |
| 47 | PEX2 | na | 18463 | -0.001 | 0.0784 | No |
| 48 | PEX14 | na | 18842 | -0.003 | 0.0680 | No |
| 49 | GSTK1 | na | 19412 | -0.008 | 0.0528 | No |
| 50 | IDH2 | na | 20417 | -0.017 | 0.0263 | No |
| 51 | MPV17L | na | 20456 | -0.017 | 0.0278 | No |
| 52 | ECI2 | na | 20472 | -0.017 | 0.0300 | No |
| 53 | SOD1 | na | 20880 | -0.021 | 0.0214 | No |
| 54 | IDH1 | na | 20925 | -0.021 | 0.0235 | No |
| 55 | PHYH | na | 21314 | -0.025 | 0.0161 | No |
| 56 | HSD17B4 | na | 21845 | -0.031 | 0.0054 | No |
| 57 | PEX1 | na | 21943 | -0.032 | 0.0074 | No |
| 58 | ABCD3 | na | 22653 | -0.039 | -0.0071 | No |
| 59 | PEX13 | na | 22983 | -0.043 | -0.0100 | No |
| 60 | DECR2 | na | 23704 | -0.052 | -0.0228 | No |
| 61 | AGXT | na | 23856 | -0.054 | -0.0189 | No |
| 62 | PIPOX | na | 24187 | -0.059 | -0.0195 | No |
| 63 | ACSL3 | na | 24235 | -0.059 | -0.0117 | No |
| 64 | ACSL5 | na | 24724 | -0.067 | -0.0156 | No |
| 65 | ACSL4 | na | 25273 | -0.075 | -0.0200 | No |
| 66 | CAT | na | 25392 | -0.077 | -0.0117 | No |
| 67 | DDO | na | 25451 | -0.078 | -0.0015 | No |
| 68 | SOD2 | na | 25515 | -0.079 | 0.0087 | No |
| 69 | AMACR | na | 25924 | -0.086 | 0.0101 | No |
| 70 | ACOX2 | na | 27125 | -0.111 | -0.0077 | No |
| 71 | PXMP2 | na | 27853 | -0.128 | -0.0092 | No |
| 72 | DAO | na | 28346 | -0.139 | -0.0021 | No |
| 73 | ABCD2 | na | 29490 | -0.172 | -0.0088 | No |
| 74 | CROT | na | 29531 | -0.173 | 0.0165 | No |
| 75 | EHHADH | na | 30010 | -0.189 | 0.0315 | No |
| 76 | ACSL6 | na | 30844 | -0.216 | 0.0404 | No |
| 77 | PEX6 | na | 31311 | -0.233 | 0.0625 | No |
| 78 | NOS2 | na | 31398 | -0.236 | 0.0961 | No |
Table: GSEA details [plain text format]

  

Fig 2: KEGG\_PEROXISOME      
 Blue-Pink O' Gram in the Space of the Analyzed GeneSet

  

Fig 3: KEGG\_PEROXISOME: Random ES distribution      
 Gene set null distribution of ES for **KEGG\_PEROXISOME**
